# A genetic risk score using human chromosomal-scale length variation can predict schizophrenia

**DOI:** 10.1038/s41598-021-97983-0

**Published:** 2021-09-22

**Authors:** Christopher Toh, James P. Brody

**Affiliations:** Department of Biomedical Engineering, University of California, Irvine, USA

**Keywords:** Computational biology and bioinformatics, Data mining, Genome informatics, Machine learning

## Abstract

Studies indicate that schizophrenia has a genetic component, however it cannot be isolated to a single gene. We aimed to determine how well one could predict that a person will develop schizophrenia based on their germ line genetics. We compared 1129 people from the UK Biobank dataset who had a diagnosis of schizophrenia to an equal number of age matched people drawn from the general UK Biobank population. For each person, we constructed a profile consisting of numbers. Each number characterized the length of segments of chromosomes. We tested several machine learning algorithms to determine which was most effective in predicting schizophrenia and if any improvement in prediction occurs by breaking the chromosomes into smaller chunks. We found that the stacked ensemble, performed best with an area under the receiver operating characteristic curve (AUC) of 0.545 (95% CI 0.539–0.550). We noted an increase in the AUC by breaking the chromosomes into smaller chunks for analysis. Using SHAP values, we identified the X chromosome as the most important contributor to the predictive model. We conclude that germ line chromosomal scale length variation data could provide an effective genetic risk score for schizophrenia which performs better than chance.

## Introduction

Schizophrenia is a highly heritable, complex psychiatric disorder^1,2^. Genome wide association studies have identified over one hundred genetic loci that contribute to its heritability^2–7^. However, these loci still account for less than half of the genetic risk for schizophrenia^3^. Environmental exposure to chemicals appears to play almost no role in the development of schizophrenia, but different forms of trauma experienced during development does appear to be a risk factor^8^. Twin studies have consistently shown a significant genetic contribution to schizophrenia, and many twin studies find that the environmental contribution to schizophrenia exists but that genetic effects provide significant liability to schizophrenia^9^.

Genetic risk scores^10–12^ have been developed for many different forms of disease, including breast cancer^13^, coronary artery disease^14^, and stroke^15^. Polygenic risk scores based on SNPs clearly can predict schizophrenia. One study measured an odds ratio of about 8 (95% CI 4–14) for the highest decile compared to the lowest decile^16^. A second study found that polygenic risk scores for schizophrenia (and bipolar disorder) are also associated with creativity^17^. A review of polygenic risk scores for schizophrenia highlighted the difficulty these studies had finding a consistent diagnosis of schizophrenia^18^. One limitation of polygenic risk scores is that they only consider linear combinations of SNPs.

Copy number variations (CNVs) in germ line DNA have also been associated with schizophrenia^4,5,19–24^. Evidence suggests that these CNVs associated with schizophrenia are represented also by SNPs^24^; the predictive power of CNVs does not add to the predictive power of SNPs when using linear prediction algorithms. The dimensionality of the data (many more SNPs than patients with schizophrenia) precludes the use of non-linear machine learning techniques.

Chromosome-scale length variation (CSLV) reduces the dimensionality of the data, while maintaining sufficient information for predictive algorithms. Combining CSLV with modern machine learning classification algorithms provides a powerful tool to predict phenotypes from a person’s genome^25^.The CSLV values are averages, across all or most of a chromosome, of copy number variation (CNV) measured at each SNP location. This method is particularly appealing for genetic risk scores because it includes epistatic effects that might be missed with conventional genome wide association studies, which use logistic regression—a linear combination of SNP scores. By attempting to still utilize every CNV value, this model aims to demonstrate that there are likely global CNV interactions which may be missed by conventional genetic risk scores.

The purpose of this paper is to evaluate how well a genetic risk score based on chromosome-scale length variation and machine learning classification algorithms can predict schizophrenia in individuals. We evaluated this approach on a dataset of 1129 patients who had schizophrenia in the UK Biobank dataset. These patients were previously genotyped as part of the UK Biobank project.

## Methods

Data was obtained from the UK Biobank under Application Number 47850. The UK Biobank project collected extensive data from about 500,000 people who were between the ages of 40 and 69 during the 2006–2010 recruitment years. This data included genotyping data and medical records. In addition, most of the participants’ medical records are linked, through the National Health Service, to the UK Biobank records. This linkage provides for ongoing follow-up of health conditions^26,27^.

First, we downloaded the “l2r” files from the UK Biobank. Each chromosome has a separate “l2r” file. Each “l2r” file contained 488,377 columns and a variable number of rows. Each column represented a unique patient in the dataset, who can be identified with an encoded ID number. Each row represented a different location in the genome. The values in the file represent the log base 2 ratio of intensity relative to the expected two copies measured at the SNP location.

After downloading the “l2r” data from the UK Biobank, we computed the mean l2r value for different portions of each chromosome for each patient in the dataset. We created three different datasets, which we refer to as “splits”. We split each chromosome into either 1, 4, or 8 nominally equal parts. Then, we compute the length for each person’s chromosome split using the l2r files by taking the average of all l2r values measured within that portion of the chromosome split. A value of 0 represents the nominal average length of that portion of the particular chromosome. We call this dataset the chromosome-scale length variation (CSLV) dataset.

The CLSV numbers represent the copy number of the genomic DNA recognized by the probe. We computed a measure of the length of chromosomes, or chromosome fragments, by averaging these l2r measurements from different probes along the chromosome. For each person, we have 1 split, 4 split, and 8 split datasets. The 1 split data consists of 23 numbers, one for each of the autosomes and one for the X chromosome. The 4 split data consists of 92 numbers and the 8 split data has 184 numbers for each person.

This CSLV dataset was matched with the UK Biobank Health records dataset. UK Biobank matched the person in the Public Health England data with UK Biobanks internal records to produce the person’s encoded participant ID. The dataset we have, provided by UK Biobank, contains the participant ID and date the patient was diagnosed by a doctor as having schizophrenia.

Using the CSLV-Schizophrenia dataset, we selected all people who had a diagnosis of schizophrenia and labelled them in the dataset. We constructed an age-matched control group of the same size that had an identical age profile as those in the schizophrenia group. The age-matched control group was selected from all those in the UK Biobank dataset having no indication of schizophrenia. Since only a small fraction of the people in the UK Biobank had a schizophrenia diagnosis, we could rerun the analysis with a different age-matched control group many times to build up statistics.

We used the H_2_O machine learning package in R^28,29^. We created 100 machine learning models that were trained to classify a person in the dataset, consisting of those who had schizophrenia and age-matched controls, based solely on their chromosome scale length variation data. Each model was trained with fivefold cross-validation. Each model had a distinct set of controls. These models were trained to perform a binary classification, distinguishing between those who had been diagnosed with schizophrenia and those who did not have schizophrenia. The models were evaluated by measuring the area under the curve of the receiver operating characteristic curve, known as the AUC.

The H_2_O package implements several common machine learning algorithms. Distributed Random Forest (drf) is based on an algorithm originally called “Extremely randomized trees”^30^. The Gradient Boosting Machine algorithm (gbm) builds regression trees in parallel^31,32^. The generalized linear model (glm) is implemented using an augmented linear model^33–35^. XGBoost is a refinement to the general Gradient Boosting Machine algorithm^36^. Ensembles are a combination of these other machine learning algorithms. This combination often provides superior results to any particular algorithm^37,38^. The H_2_O package implements stacked ensembles as super learner algorithms^39^. The H_2_O package also uses SHAP values to interpret the models^40^. SHAP values are measures of how important different features are to the prediction.

Our computer analysis system is a Linux server running Ubuntu 18.04. The system is a 64-bit system running two Intel Xeon E5-2690 2.90 GHz CPUs. It also has a GeForce GT 710 NVIDIA GPU. 32 GBs of RAM were also available with a 10 TB HDD.

### Ethics approval and consent to participate

Ethics approval and participant consent was collected by UK Biobank at the time participants enrolled. All subjects in the database have given informed consent, and if under 18, consent from a parent and/or legal guardian. Additionally, all subjects have the ability to withdraw at any time from the UK Biobank. This paper is an analysis of anonymized data provided by UK Biobank. According to UC Irvine’s IRB, analysis of anonymized data does not constitute Human Subjects Research. All methods and experimental research protocols were approved by the UK Biobank.

## Results

**Figure 1:**
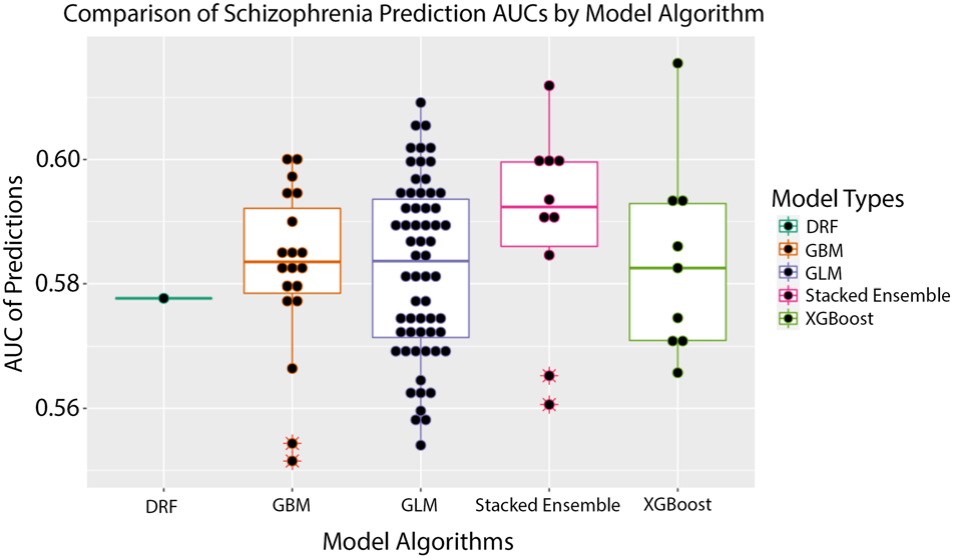
This boxplot figure presents the results of the machine learning predictions. We created 100 different datasets. For each dataset, we used the same set of schizophrenia patients with a distinct set of age matched people from the general UK Biobank population as controls. Then H2O was used to perform a grid-search of possible best algorithms. The best performing algorithm was then reported with an AUC. The differences between algorithms is reported here. The machine learning algorithms tested were distributed random forests (drf), gradient boosting machine (gbm), general linear model (GLM), stacked ensemble (a combination of the other four algorithms) and XGBoost (XGBoost).

Figure 1 presents results showing the performance of different machine learning algorithms. We found that the stacked ensemble models consistently performed best. As Fig. 1 shows, we found a slight difference between algorithms and their performance. But all algorithms could predict schizophrenia significantly better than chance (AUC = 0.50). This finding indicates that germ line genetics of the patient, as represented by the set of chromosome-scale length variation numbers, demonstrates predictability of schizophrenia.

The AUC (area under the curve of the receiver operating characteristic curve) for the machine learning classification models was 0.583 (standard deviation 0.014, 95% confidence interval of 0.581–0.586). A classification model with an AUC of 0.50 is equivalent to random guessing. The measured AUC differs from 0.50 with p < 0.00001.

We also tested how well each model could predict schizophrenia on a holdout set of validation data. The holdout set was 30% of the original test data and was not included in the training of the models. The AUC of the holdout set was 0.5734 with a 95% confidence interval of 0.569–0.578.

We then tested whether increasing the number of splits improves model performance. We constructed three overlapping datasets with 1 split, 4 splits, and 8 splits. The phrase “1 split” represents the average l2r value measured across an entire chromosome for all 23 chromosomes giving a total of 23 numbers, “4 splits” represents the average of each quarter of the 23 chromosomes l2r values for a total of 92 numbers, and “8 splits” represent the average of each eighth of the 23 chromosomes’ l2r values for a total of 184 numbers.

**Figure 2:**
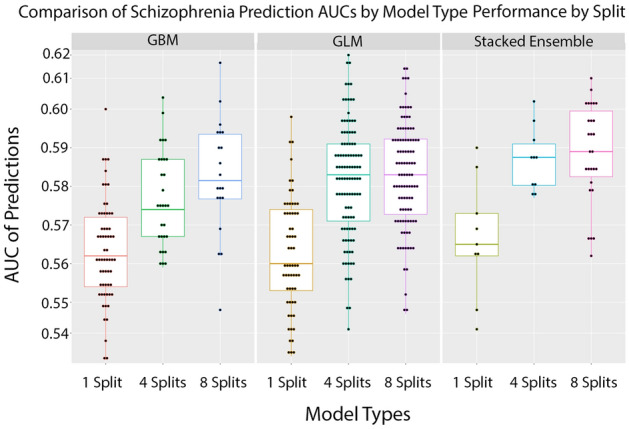
We tested whether finer splits of each chromosome lead to better predictability. We split each chromosome into either one, four, or eight subsections. We computed the chromosome scale length variation for each of these subsections for each person. This set of numbers was used to predict whether patients had schizophrenia. The quality of this prediction was characterized by the AUC. This plot demonstrates how the quality of these predictions increase with finer information on chromosome length variation. The Stacked Ensemble algorithm performs the best across all split variations.

Figure 2 shows how models compare on the 3 different split datasets. Overall, a stacked ensemble had the best performance, however a general linear model (glm) was most often the best candidate model.

**Figure 3:**
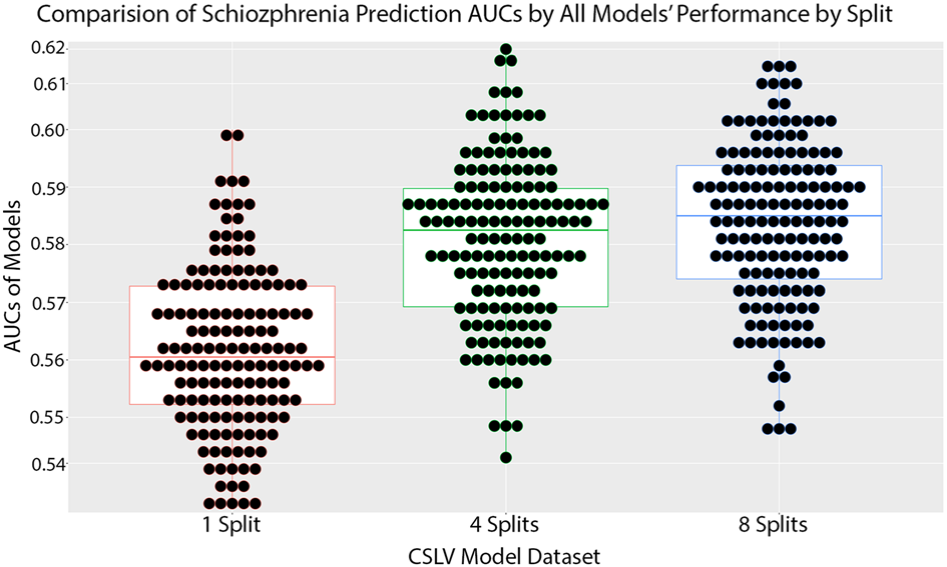
This plot represents the average performance of 150 models for each split type for a total of 450 models.

In all models, increasing splits improves model performance for the same runtime. Figure 3 demonstrates the difference of all models for 1 split, 4 splits, and 8 splits datasets. We tested whether finer splits of the dataset provided significantly improved AUCs. As shown in Table 1, the p-value of the 4 splits model compared to the 1 split model is $p=1\times {10}^{-24}$. Comparing the mean AUC for the 8 splits model to the 1 split model gave a p-value of $p=3\times {10}^{-30}$ indicating that finer splits significantly improved the predictive ability of the models. The 4 splits and 8 splits models performed better than the 1 split models by a significant amount.

**Table 1:** The mean and standard deviation of the cross validated AUCs of 1 split, 4 splits, and 8 splits datasets of 150 models each.

Dataset	Mean AUC	Standard Deviation	P-value vs 1 split
1 split	$0.5614$	$0.0148$	
4 splits	$0.5807$	$0.0146$	$1\times {10}^{-24}$
8 splits	$0.5838$	$0.0141$	$3\times {10}^{-30}$

We then calculated the odds ratio (OR) of our predictions drawn from the cross-validated model. Table 2 shows that a patient in the upper quintile is approximately twice as likely to have schizophrenia when compared to the lower quintile.

**Table 2:** This table represents the odds ratio between the quintiles of predicted results from our cross-validated results. The result indicates that the top quintile is twice as likely to have an accurate prediction for Schizophrenia as the bottom quintile.

Quintile	Normal	Schizophrenia	Odds ratio	Count	95% CI
1	185	123	0.67	308	0.51–0.85
2	156	152	0.97	308	0.76–1.24
3	153	155	1.0	308	0.79–1.3
4	142	165	1.2	307	0.91–1.5
5	133	174	1.3	307	1.0–1.7

**Figure 4:**
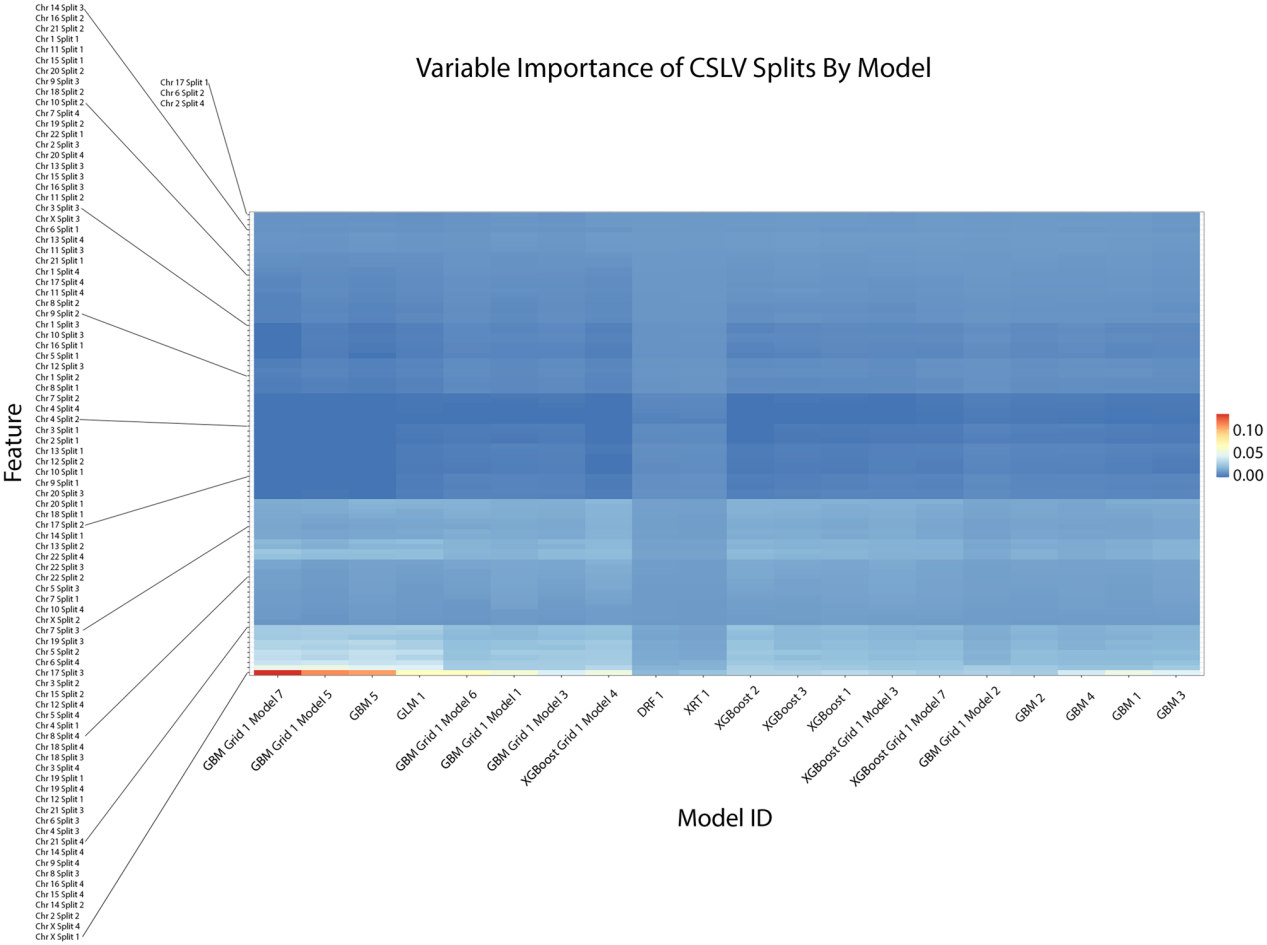
This variable importance heatmap shows the variables which most affected the performance and outcome of decisions made by the specified model. A value closer to 1.0 indicates higher importance of that variable. In most tree-based models the CSLV values for chromosome X have the highest importance.

**Figure 5:**
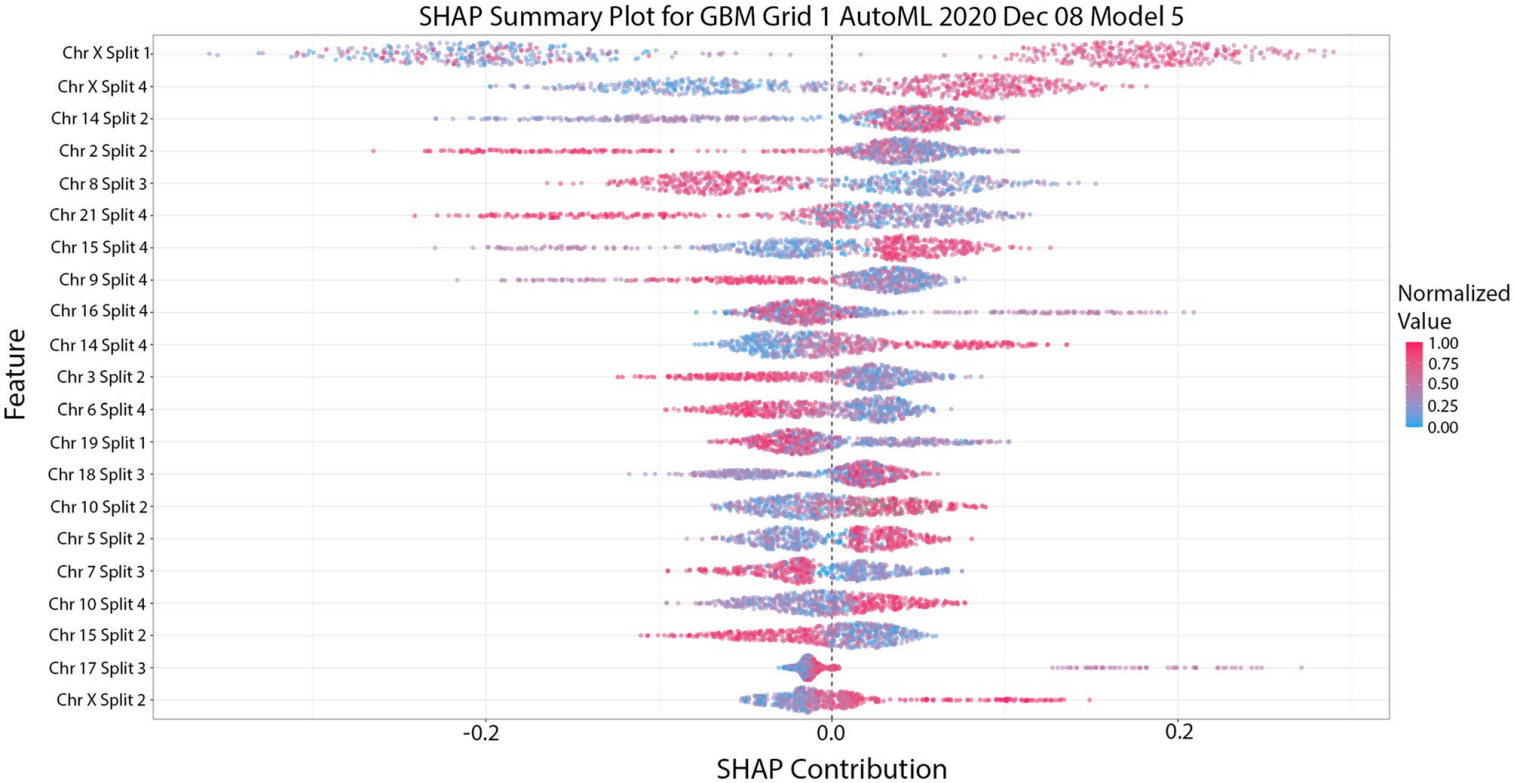
This SHAP plot indicates that the leading model for our 4-splits model relied heavily on the first quarter and last quarter value of chromosome X with some contribution from other regions and the second quarter of chromosome X.

In order to understand how our models came to their conclusions, we created several plots to explain them from H2O’s “explainability” framework. The first is a variable importance heatmap across the generated models which is shown in Fig. 4. Our analysis here indicated that chromosome X was one of the highest contributing variables in predicting Schizophrenia, especially in tree models such as GBM and XGBoost. We then confirmed this with a Shapley Additive exPlanation or SHAP plot in Fig. 5. This plot also indicates that chromosome X was the leading factor in our leading model for predicting schizophrenia.

**Figure 6:**
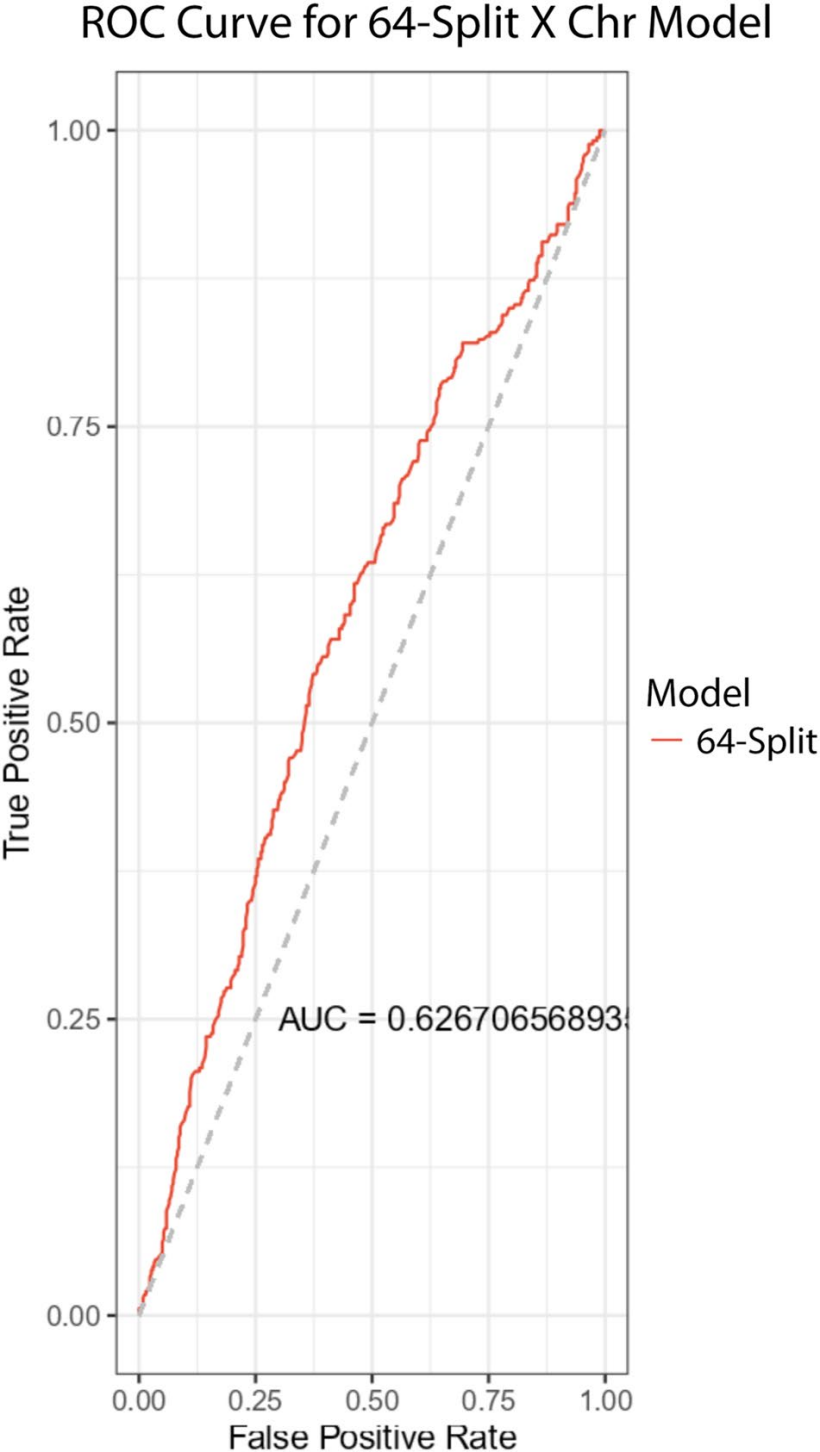
This ROC Curve for a schizophrenia prediction model utilizing 64-splits or 64 CSLVs of chromosome X only. The reported AUC is 0.627.

Utilizing our findings above, we then proceeded to train new models from scratch using only CSLV values from chromosome X but with 64 CSLV splits. This model did not contain any information from the 22 autosomes but instead relied solely on CNVs in the X chromosome and our aim was to see if the model would be comparable to our previous 4-split and 8-split models. We found that on average these models had a comparable performance of about 0.58 with the highest being around 0.627 as shown in Fig. 6.

**Figure 7:**
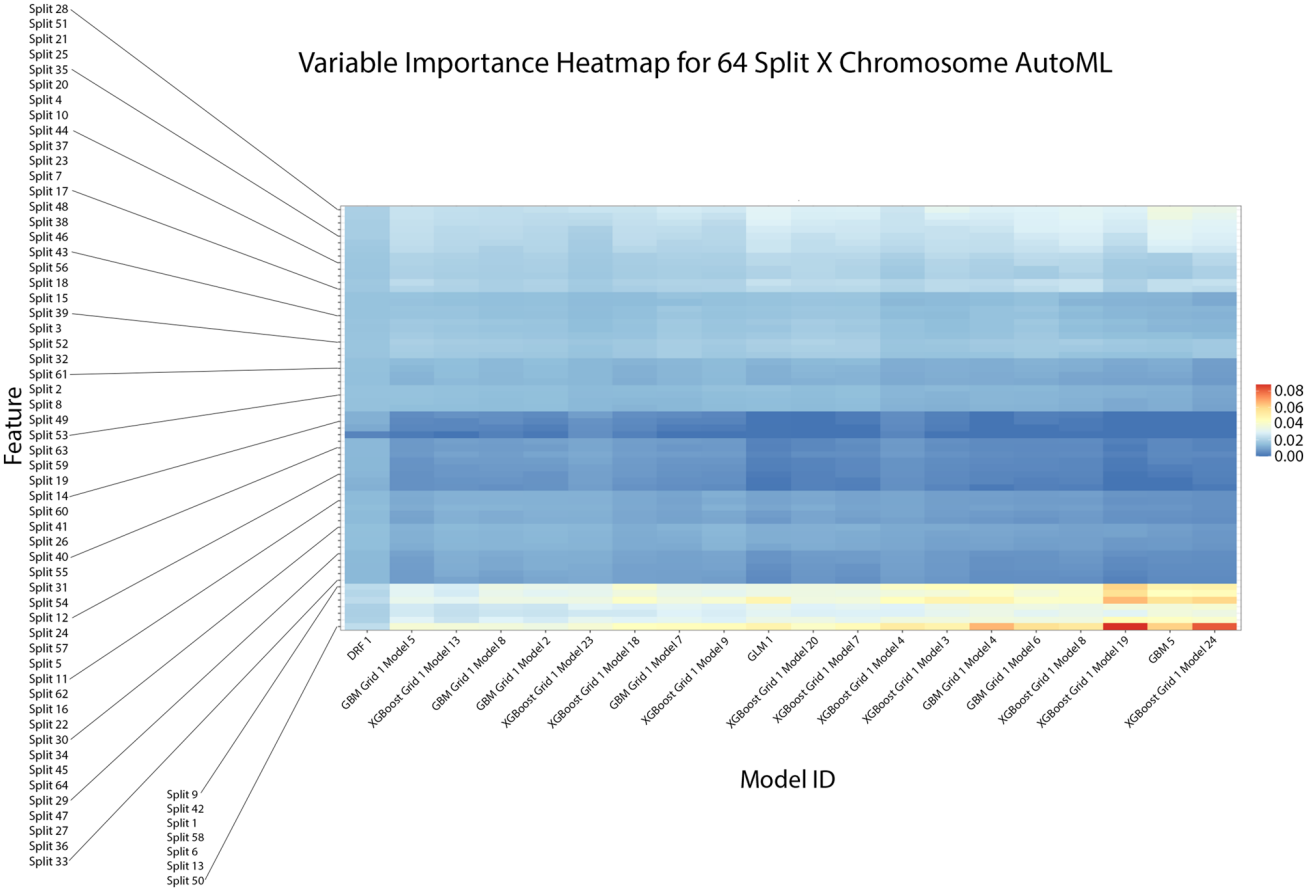
This variable importance heatmap shows the variables which most affected the performance and outcome of decisions made by the specified model. A value closer to 1.0 indicates higher importance of that variable. In most of the models we find that the CSLV values were mostly centered around split 50, 1, 9, 42, 13, 58, and 6. This is consistent with Fig. 4.

We then again performed a variable importance heatmap analysis to get greater granularity of our understanding of the contributing CSLVs in chromosome X. We found that this was again consistent with the previous findings from the 4-split model. Figure 7 indicates that the top features of variable importance are again being found in the first and last regions of chromosome X. As such it appears that the majority of the predictive power of any model trained with CSLV and when predicting schizophrenia in an individual is a result of CNVs on chromosome X. We also report corresponding estimates of hg38 coordinates in Table 3.

**Table 3:** This table shows the estimated hg38 coordinates for the corresponding CSLV splits with high variable importance as shown in Fig. 7.

CSLV Split	Estimated hg38 Coordinates
1	chrX:60425–634774
6	chrX:5651118–7792613
9	chrX:11426091–13234434
13	chrX:20912585–22990332
42	chrX:107331058–110669244
50	chrX:128031497–130523635
58	chrX:145709120–147908169

We wanted to ensure these results were not due to inherent sex differences. We trained 50 models using the 64 split chromosome X dataset which were not only age-matched with the controls but also sex-matched. 25 of the AutoML models were trained with the actual data with correctly labeled disease states. The other 25 AutoML models were trained with the schizophrenia diagnosis randomly shuffled. The results are shown in Table 4. Here we can see that a portion of the previous performance is most likely due to CSLV differences inherent between males and females (Supplemental D). However, a portion of the prediction is statistically still better than random guessing.

**Table 4:** This table shows a comparison of the age and sex matched models using 64 Split chromosome X data. The reported mean AUCs demonstrates that a portion of the previous performance is attributed to differences between male and females in X Chromosome CSLV levels as shown in Supplementary Information D. However, it still performs better than randomly guessing.

Dataset	Mean AUC	Standard Deviation
64 Split × normal	0.545	0.01373103
64 Split × random	0.525	0.01363745
Welch two sample t-test between normal and random	T = − 5.0111 df = 47.998	p-value = 7.763e-06

## Discussion

These results indicate that germline genetic variation contributes at least to some degree to the onset of schizophrenia in individuals. Our results indicate that genetic structural variation across the global chromosomal scope is sufficient to predict, better than guessing, whether or not an individual will have schizophrenia. The patients were an equal number of patients by gender between the control and disease group and the ages of patients in the control group also were matched to the ages of patients in the disease group. Further analysis revealed that length variation in a handful of regions of the X chromosome was sufficient to reproduce the predictive model. Recently, there has been revived discussion of copy number variations as a large contributing factor to several neurological ailments including schizophrenia^41^. Additionally, hypotheses about sex chromosome links to schizophrenia inheritance have been discussed for several decades and our findings lend support to this idea^42^.

On average, a stacked ensemble is the best approach to creating a predictive model for the prediction of schizophrenia. However, all models that were tested still created models with predictive power better than chance (Supplementary Information A, B, & C). Since H_2_O’s AutoML performs a grid-search of all the possible datasets and each trial we ran included the same disease group but with a different control groups, we can see in Fig. 1 that a general linear model (GLM) oftentimes was still the best option. Gradient Boosted Machines (GBM) and XGBoost also typically performed the same as GLM.

Utilizing a more granularized dataset by splitting the autosomes into quarters and eighths performs significantly better than using a CSLV averaged across an entire chromosome. This observation suggests we can increase performance by increasing splits. In the future, we plan on exploring the trade off in run time and computational resources required by increasing splits. Other methods of dimensionality reduction may also yield better results without sacrificing runtime performance.

The CSLV values are averages of copy number variation (CNV) measured at each SNP location. Simply using every single CNV value introduces a dimensionality problem as our dataset only has roughly 488,000 individuals while the total number of CNV values is 764,257 across the 22 autosomes and an additional 18,857 CNV values for the X Chromosome. This means there is likely diminishing returns for using more splits unless it can be offset with increased data.

This approach has several limitations. First, CSLV is an averaged measure of copy-number variations across a large section of the entire chromosome. We used SHAP values to highlight the regions that seem to be more important, but this does not provide a mechanistic explanation. Second, the dataset lacks diversity. The UK Biobank population is primarily Caucasian individuals in the United Kingdom (although not exclusively). Third, the diagnosis of schizophrenia in an individual is difficult to quantify and the disease might consist of a heterogeneous group of underlying biological processes. Finally, this analysis is based on a single dataset and the conclusions would be stronger if the analysis could be replicated in an independent dataset. However, similar datasets are not currently available.

## Conclusion

We were able to create machine learning models for prediction of schizophrenia in patients. These models perform better than chance with an average AUC of 0.545. Prediction was performed with only chromosomal scale length variation measurements as the input variables. Further analysis of the SHAP values suggests that the length variation of several regions of the X chromosome are sufficient to reproduce this predictive value.

## Supplementary Information

Supplementary Information.

## Data Availability

The datasets analyzed during the current study are available from UK Biobank at https://www.ukbiobank.ac.uk/.

## Abbreviations

AUC: Area under the curve
CNV: Copy number variation
CSLV: Chromosomal-scale length variation
GBM: Gradient boosted machines
GLM: General linear model
ROC: Receiver operator curve
SHAP: SHapley Additive exPlanations
SNP: Single nucleotide polymorphism
