# Supplementary material for: A genetic risk score using human chromosomal-scale length variation can predict schizophrenia
Source: Sci Rep. 2021 Sep 22;11:18866. doi: 10.1038/s41598-021-97983-0 (PMC8458522; doi:10.1038/s41598-021-97983-0)
Supplement: Supplementary file 1 — Supplementary Information. [file 41598_2021_97983_MOESM1_ESM.pdf]

Supplemental:

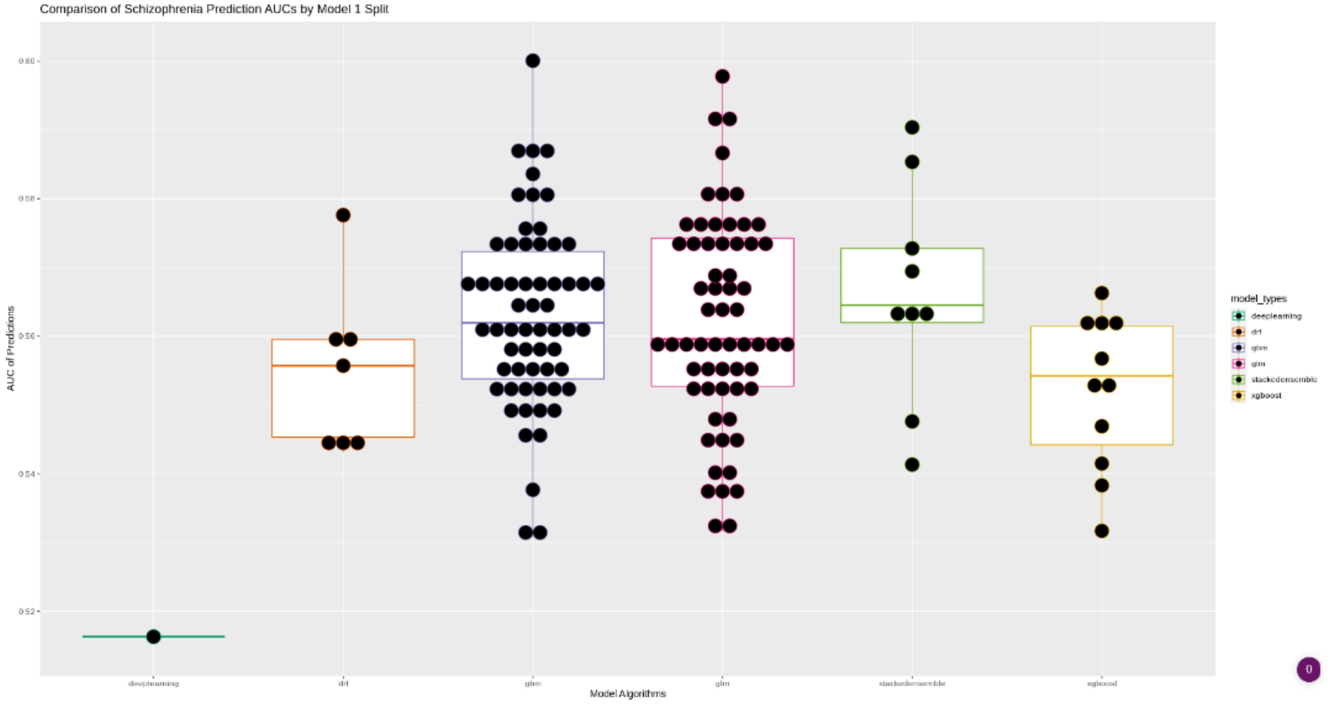

Supplemental A. This plot represents the average performance of 150 models for 1 split AutoML models divided by algorithm of the leader. In this experiment we found that overall Stacked Ensemble performs the best but most often GBM or GLM were the leading model types for an AutoML.

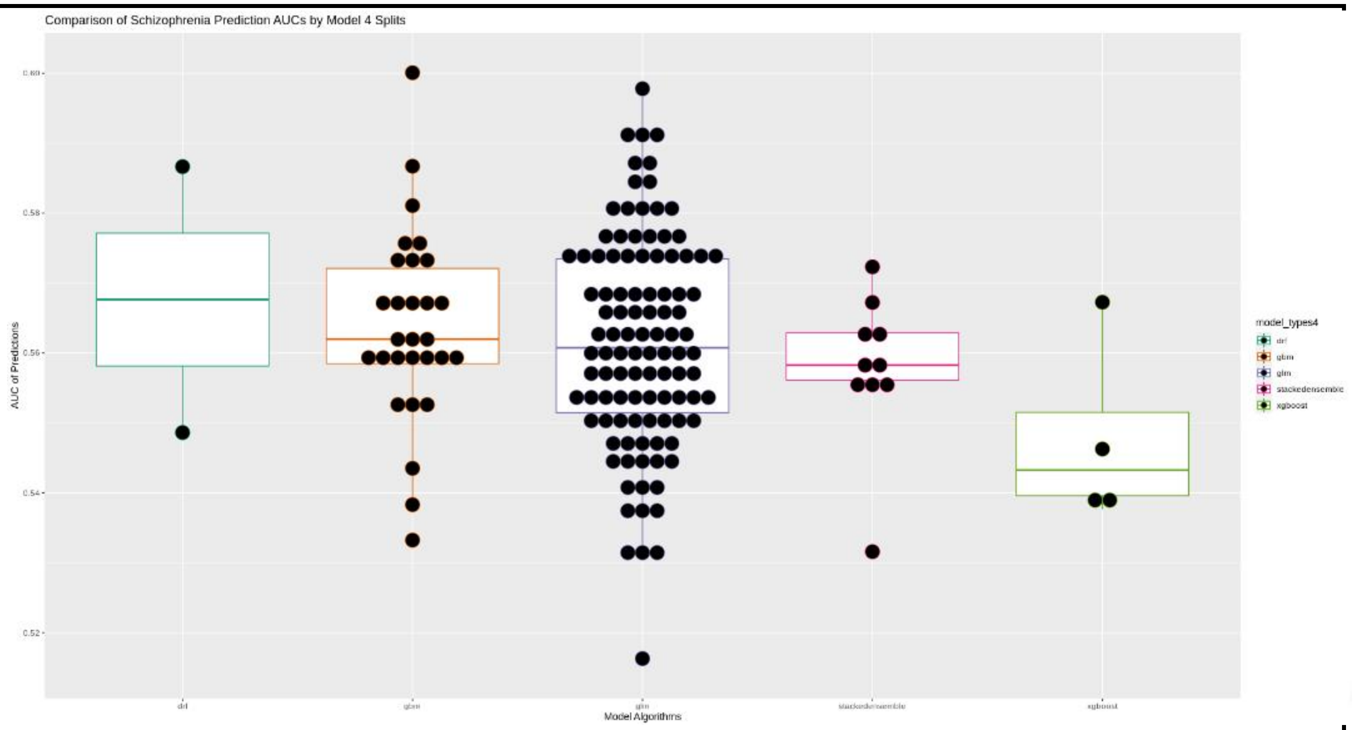

Supplemental B. This plot represents the average performance of 150 models for 4 splits AutoML models divided by algorithm of the leader. In this experiment DRF was the outlier but GLM was still the leading model in the majority of the AutoML runs. Stacked Ensemble and GBM were comparable.

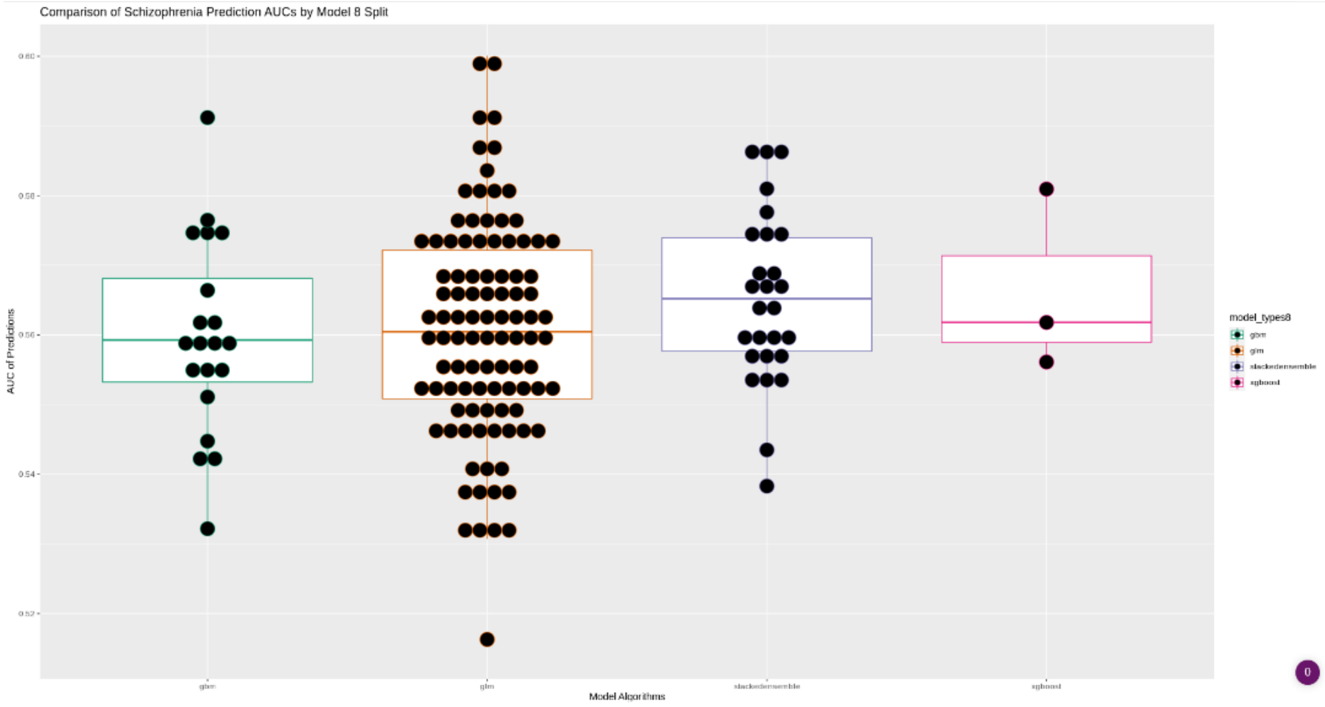

Supplemental C. This plot represents the average performance of 150 models for 8 splits AutoML models divided by algorithm of the leader. In this particular experiment Stacked Ensemble was again the best overall performing model with GLM still being the most common leader model in an AutoML. DRF was never a leader in any of these runs.

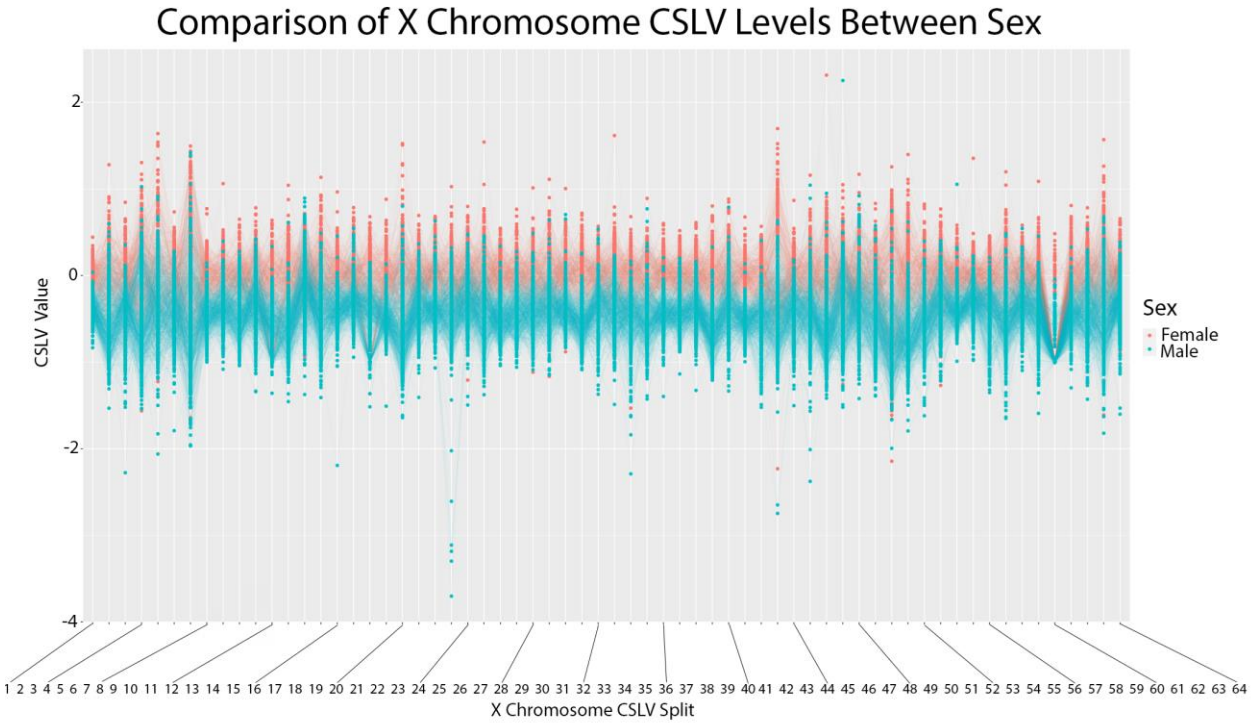

Supplemental D. This plot compares the X Chromosome CSLV values of schizophrenia patients by sex. It contains all patients from the UK Biobank with a diagnosis of schizophrenia. In general females have higher average CSLV values than males. This is consistent with the fact that females have two X chromosomes while males only have one X chromosome.
